# Supplementary material for: Comparative Proteomic Analysis of Drug Trichosanthin Addition to BeWo Cell Line
Source: Molecules. 2022 Feb 28;27(5):1603. doi: 10.3390/molecules27051603 (PMC8911981; doi:10.3390/molecules27051603)
Supplement: Supplementary file 1 [file molecules-27-01603-s001.zip › molecules-1513601supp.pdf]

# Supplementary Materials

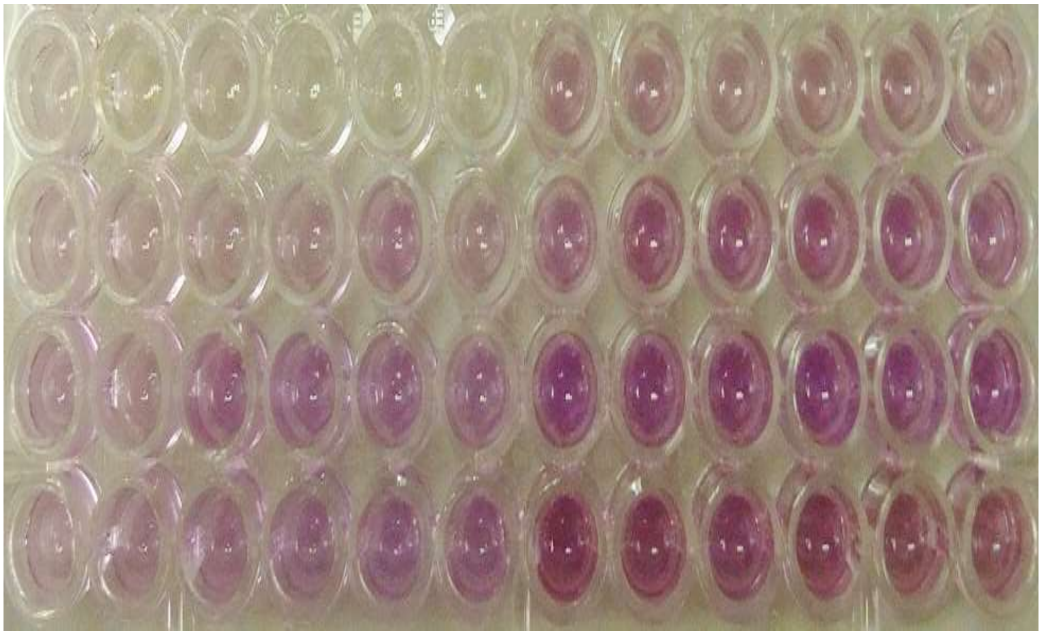

**Figure S1.** MTT result overview.

**A**

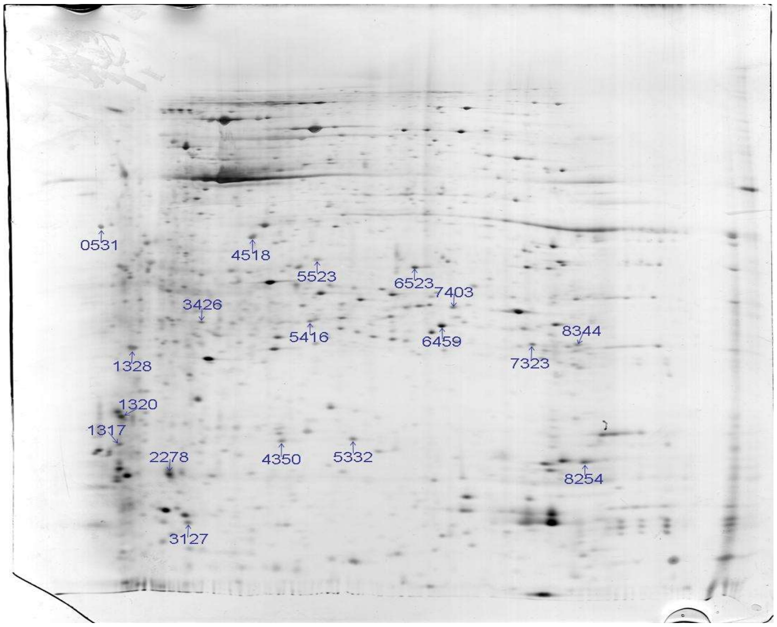


**B**

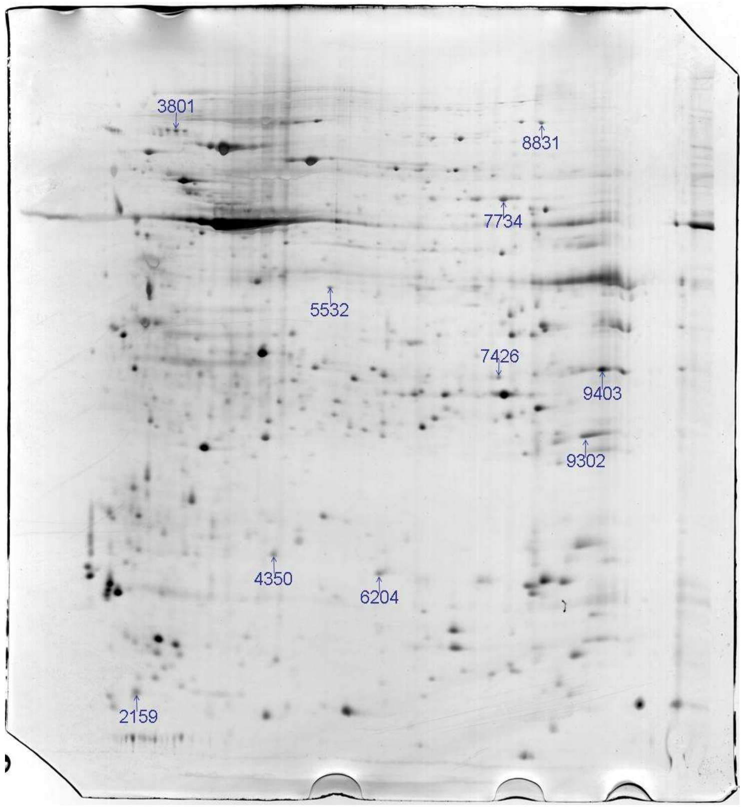


**Figure S2.** 2DE graph, (A) first repetition; (B) second repetition.. 250 ug, pH3-10 NL, 18cm, silver stained. DEPs were selected for protein identification.

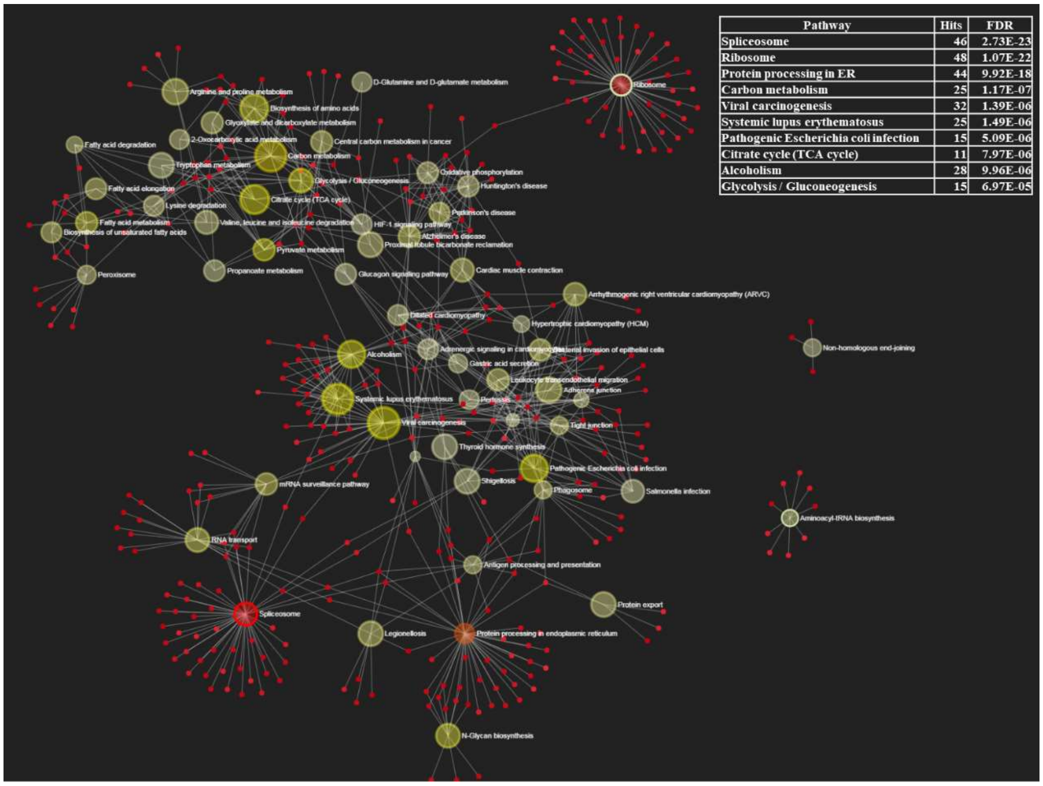


| Pathway | Hits | FDR |
|---|---|---|
| Spliceosome | 46 | 2.73E-23 |
| Ribosome | 48 | 1.07E-22 |
| Protein processing in ER | 44 | 9.92E-18 |
| Carbon metabolism | 25 | 1.17E-07 |
| Viral carcinogenesis | 32 | 1.39E-06 |
| Systemic lupus erythematosus | 25 | 1.49E-06 |
| Pathogenic Escherichia coli infection | 15 | 5.09E-06 |
| Citrate cycle (TCA cycle) | 11 | 7.97E-06 |
| Alcoholism | 28 | 9.96E-06 |
| Glycolysis / Gluconeogenesis | 15 | 6.97E-05 |

**Figure S3.** The differential expressed proteins were enriched in function-Gene biparties network by network analyst.

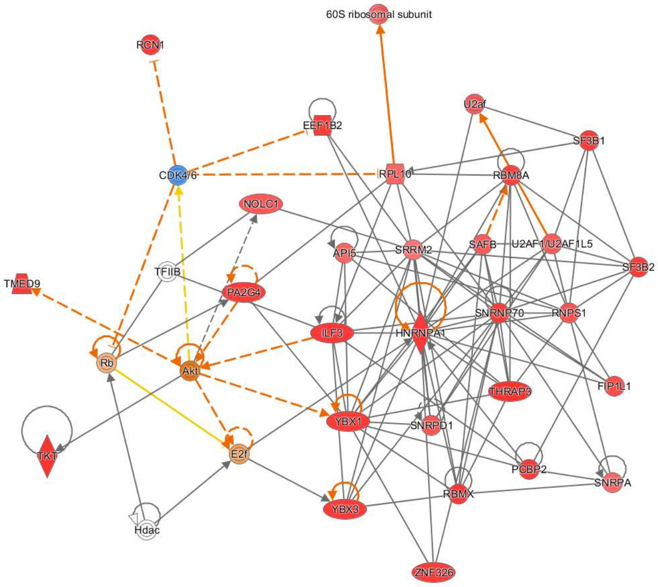


**Figure S4.** The first network of differential proteins enriched in IPA software were displayed. Among them, hub molecules Akt, CDK4 / 6, Rb, E2F were associated with tumor.

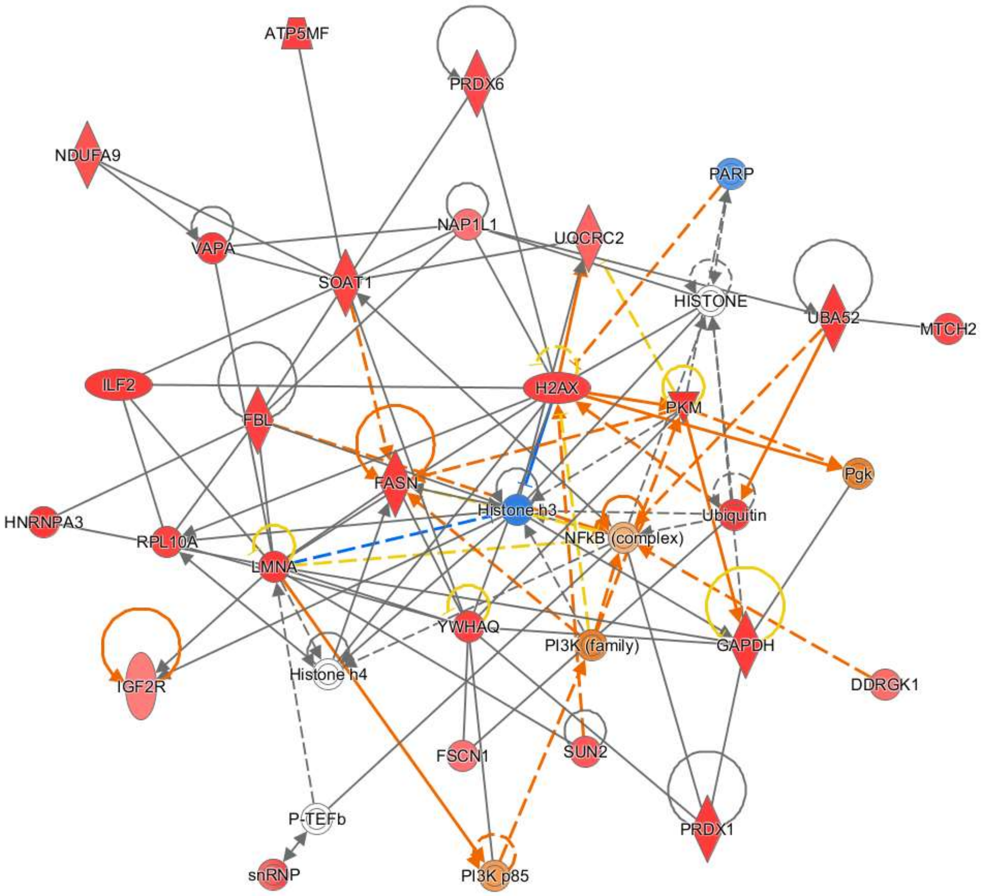


**Figure S5.** The second network of differential proteins enriched in IPA software were displayed. Among them, hub molecules FASN, Histone h3, H2AX, EMNA, FBL, SOAT1, PKM, PI3K, NFKB, PI3K were associated with tumor.

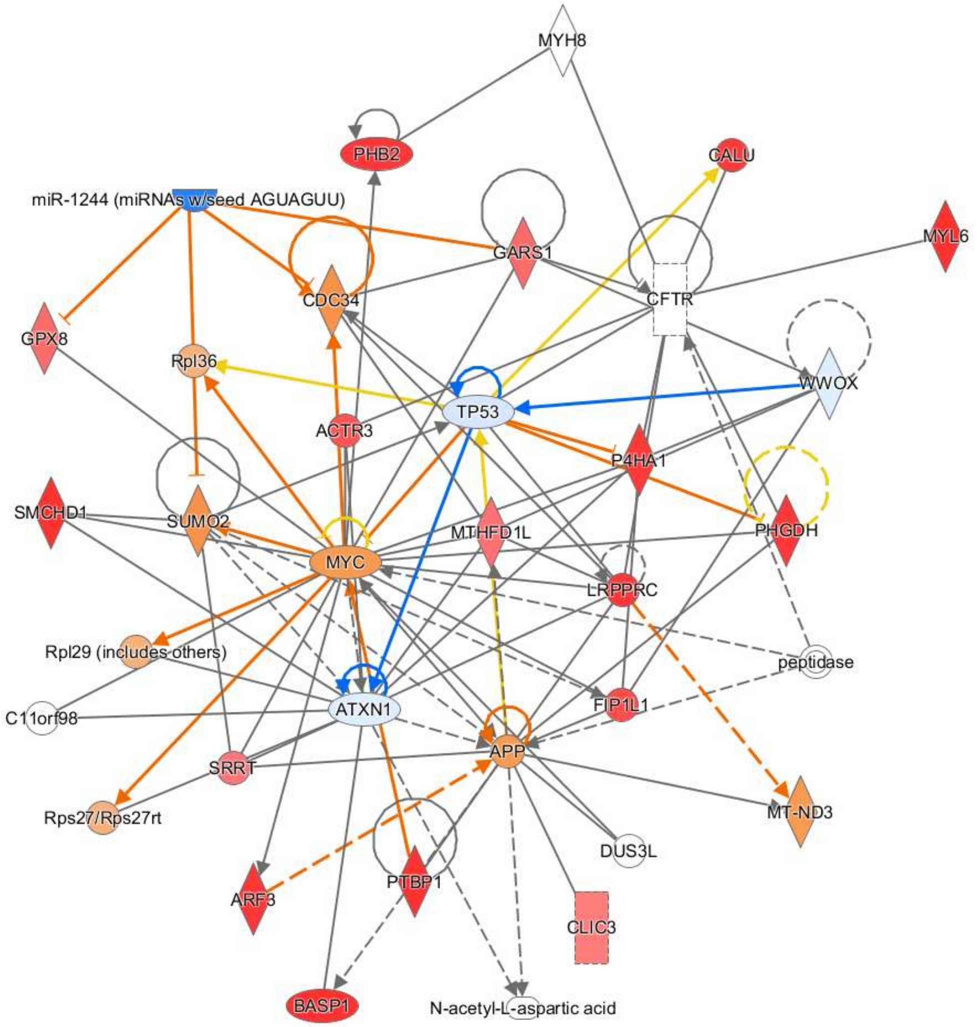


**Figure S6.** The third network of differential proteins enriched in IPA software were displayed. Among them, hub molecules MYC, ACTR3, TP53, ATXN1 were associated with tumor.

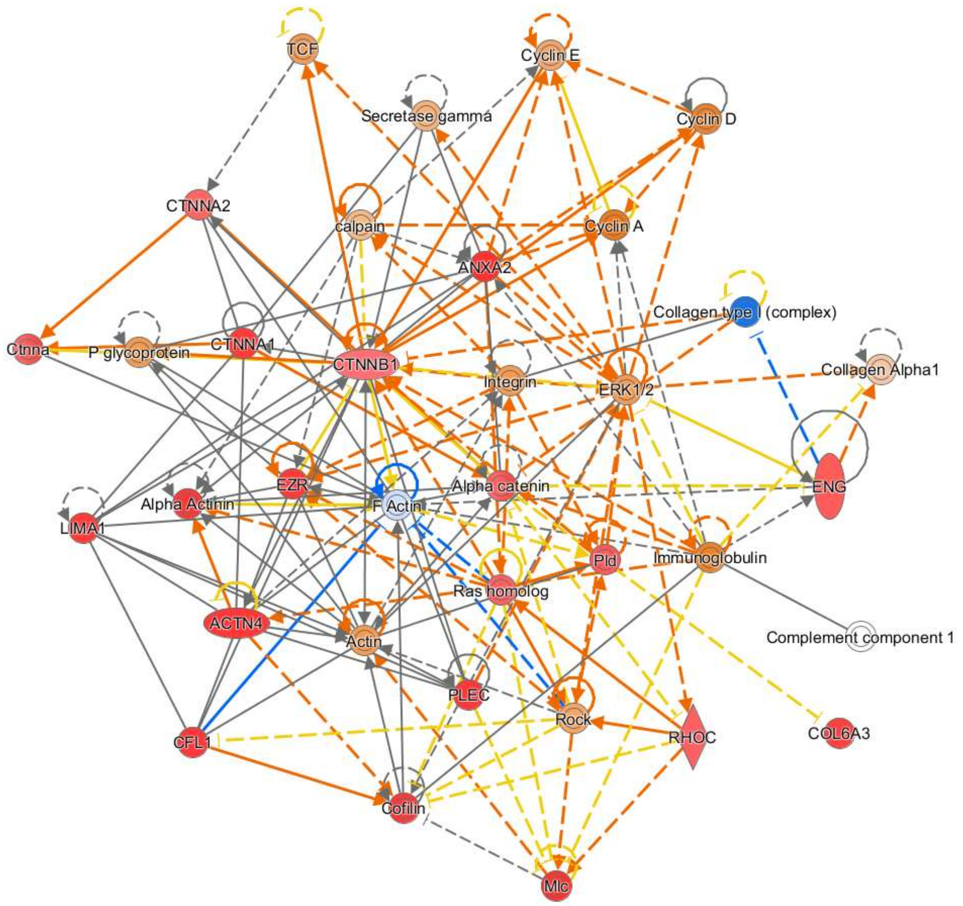


**Figure S7.** The fourth network of differential proteins enriched in IPA software were displayed. Among them, hub molecules CTNNB1, ERK, F Actin, CTNNA2 were associated with tumor.

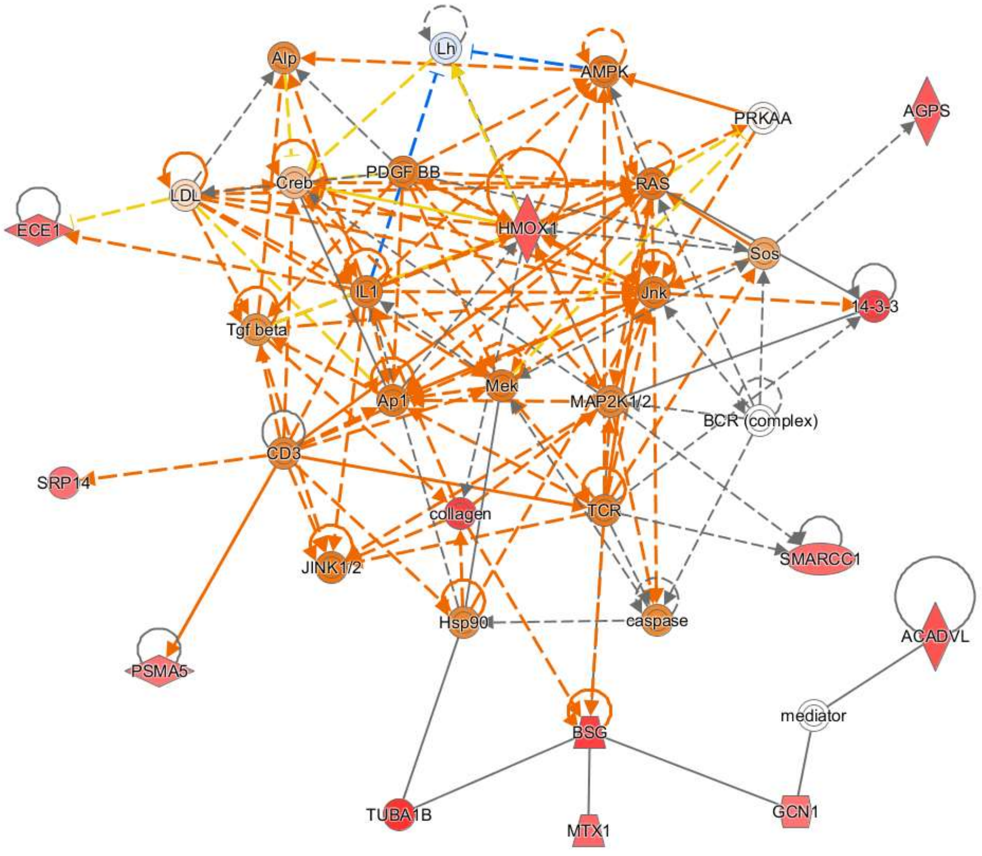


**Figure S8.** The fifth network of differential proteins enriched in IPA software were displayed. Among them, hub molecules PDGF BB, HMO X1, L1, MAP2K, TCR were associated with tumor.

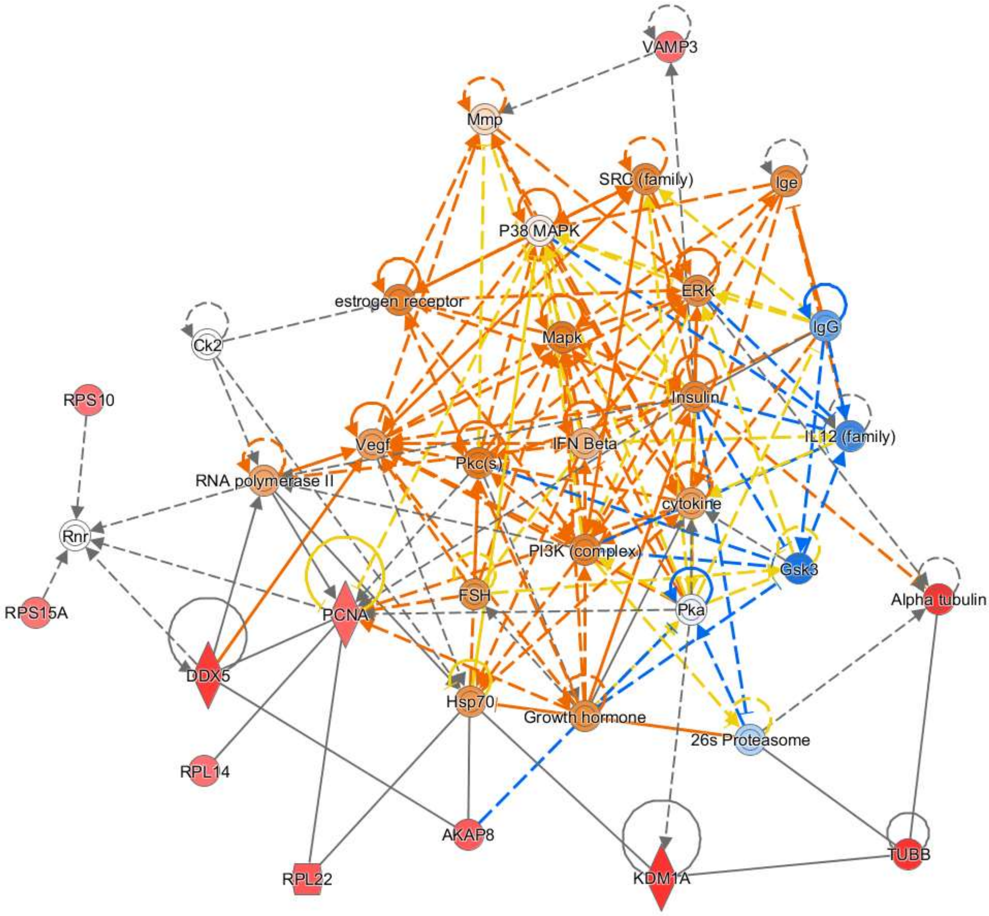


**Figure S9.** The sixth network of differential proteins enriched in IPA software were displayed. Among them, hub molecules Insulin, IFN Beta, pkc, FSH, Hsp70, Mapk, ERK were associated with tumor.
